# Seasonal variation of idiopathic benign paroxysmal positional vertigo correlates with serum 25-hydroxyvitamin D levels: a six-year registry study in Shanghai, China

**DOI:** 10.1038/s41598-019-52803-4

**Published:** 2019-11-07

**Authors:** Liang Shu, Jing Wu, Chun-Yan Jiang, Xu-Hong Sun, Hui Pan, Jie Fang, Yi Tang, Si-Cheng Wu, Jian-Ren Liu, Wei Chen

**Affiliations:** 1 Department of Neurology, Shanghai Ninth People’s Hospital, Shanghai Jiao Tong University School of Medicine, Shanghai, China; 2 Department of Clinical Laboratory, Shanghai Ninth People’s Hospital, Shanghai Jiao Tong University School of Medicine, Shanghai, China; 3 Biostatistics Office of Clinical Research Center, Shanghai Ninth People’s Hospital, Shanghai Jiao Tong University School of Medicine, Shanghai, China

**Keywords:** Risk factors, Neurological disorders

## Abstract

Seasonal variation of benign paroxysmal positional vertigo (BPPV) occurrence has been reported in recent years. Whether the seasonality of BPPV also exists in Chinese patients and whether it correlates with serum vitamin D levels is unexplored. We retrospectively analyzed the data of 1269 new-onset idiopathic BPPV patients registered in our vertigo outpatient clinic over a six-year period. Additionally, serum 25-hydroxyvitamin D levels during this period were measured in 877 patients by chemiluminescence immunoassay. We delineated the changing trend of the monthly BPPV patient numbers and serum 25-hydroxyvitamin D levels, and the correlation between them was explored. December to next March is the top 4 months with higher BPPV patient numbers. The median BPPV patient numbers in winter group were higher than those in summer group (20 vs. 16 patients, *p* < 0.05). Median 25-hydroxyvitamin D levels in winter group were much lower than those in summer group (16.3 vs. 20.8 ng/ml, *p* < 0.001) and autumn group (16.3 vs. 19.3 ng/ml, *p* < 0.05). A moderate negative correlation was observed between median serum 25-hydroxyvitamin D levels and BPPV patient numbers each month. The onset of BPPV also shows a seasonal fluctuation in Chinese patients. This phenomenon may be related to serum vitamin D levels.

## Introduction

Benign paroxysmal positional vertigo is the most common peripheral vestibular disease, with a reported prevalence of 10.7 to 64.0 cases per 100,000 population in the USA and a lifetime prevalence of 2.4% in Germany^1,2^. Pathologically, it is caused by dislodged otoconial debris that moves from the utricle to the semicircular canals. The underlying mechanism is much more complicated: aging, otolithic degeneration, abnormal bone metabolism, low vitamin D levels, higher uric acid, and hormonal changes represent predisposing factors for idiopathic BPPV^3–9^.

A seasonal variation has been observed among patients presenting with BPPV in the United States, Iraq, and the UK in recent years^10–12^. However, the underlying cause remains uncertain. Varying vitamin D levels were influenced by climatic factors, such as temperature, daily sunlight, and ultraviolet index, which may correlate with the seasonality of BPPV occurrence. However, no direct evidence to confirm this hypothesis has been shown yet. Shanghai is located in eastern China without high altitude. It is a coastal city with subtropical monsoon climate and distinct four seasons. As the location and climate in Shanghai is different from the region that previous studies were carried out, whether Chinese patients in Shanghai also present a seasonal feature deserves investigation.

Therefore, the present study first validated whether there was a seasonal change in Chinese BPPV patients registered within six years in our clinical center, and secondly, explored whether it was related to variations in serum vitamin D levels.

## Methods and Materials

### Patients

We retrospectively reviewed baseline records of BPPV patients presenting to our vertigo outpatient clinic from March 2013 to February 2019. Four authors (L.S., J.W., Y.T. and W.C.) collected the original data together. A diagnosis of BPPV was based on (1) a history of a brief episode of vertigo induced by head motion, (2) a typical positioning nystagmus characteristic of BPPV, which was induced by Dix-Hallpike or supine roll test^13,14^, and (3) no other identified disorders of the central nervous system. Every BPPV patients underwent canalith repositioning (CRP) maneuvers right after diagnosis. When the symptom was not relieved through repeated CRP maneuvers (no more than three times) and positional nystagmus persisted, brain imaging was performed to rule out any central pathological conditions. We included idiopathic BPPV patients. Patients with secondary BPPV, such as head trauma, vestibular neuritis, vestibular migraine and Meniere disease were excluded. Only the first diagnosis of idiopathic BPPV in our clinical center was recorded even if the patient experienced a recurrence within this six-year period. The study was approved by the Ethics Committee of Shanghai Ninth People’s Hospital, Shanghai Jiao Tong University School of Medicine, Shanghai, China. Written informed consent was obtained from each participant according to national and institutional guidelines.

### Clinical and biochemical profile

Demographic data, including age, gender, sleeping habits, concomitant diseases, time period of symptom onset to confirmed diagnosis and date of diagnosis, were recorded for each patient. According to the Chinese lunar calendar and climate, one year in Shanghai could be categorized into four seasons: spring (March to May), summer (June to August), autumn (September to November), and winter (December to the following February). All idiopathic BPPV patients were divided into four groups according to different onset seasons: spring, summer, autumn and winter group. In addition, venous blood was collected from most registered patients soon after CRP therapy on the same day. As reported, serum 25-hydroxyvitamin D levels were measured by chemiluminescence immunoassay (Diasorin Inc, USA)^3,15^.

### Statistics

Statistical analysis was conducted using GraphPad Software 7 (GraphPad Software Inc. La Jolla, CA 92037, USA) and SPSS 23 (IBM SPSS software Inc., Chicago, IL, USA). Graphs were delineated by using GraphPad Software 7. All continuous variables were first tested for normality using the Kolmogorov-Smirnov test. Variables with a normal distribution were recorded as mean ± SD and were tested by an independent two-tailed *t*-test. The Mann-Whitney *U* test was chosen for non-normally distributed data. Categorical variables presented in absolute numbers were assessed using the Chi-squared test or Fisher’s exact test (two-tailed). Multiple comparisons of BPPV patient number and serum 25-hydroxyvitamin D value among different four groups were conducted by using one-way ANOVA Post Hoc test (Scheffe method) after homogeneity of variance test. To explore the correlation between pooled BPPV patient numbers and serum 25-hydroxyvitamin D levels, a non-parametric Spearman correlation test was performed. Moderate correlation refers to *r* value ranging from 0.5 to 0.8. Statistical significance was defined as *p* < 0.05.

### Ethical approval

All procedures were in accordance with the ethical standards of the responsible committee on human experimentation and with the Helsinki Declaration.

## Results

### Idiopathic BPPV patient numbers

**Figure 1:**
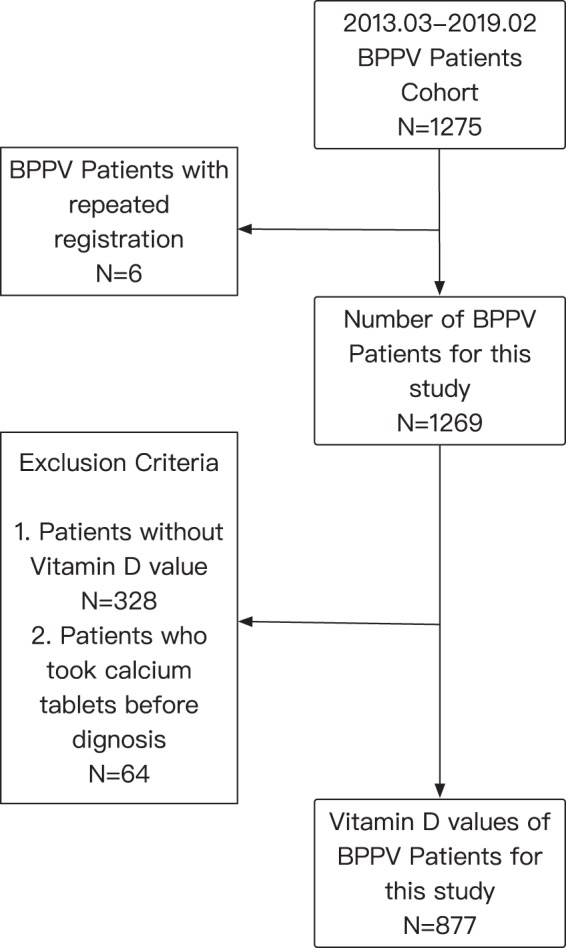
Patient Selection.

**Figure 2:**
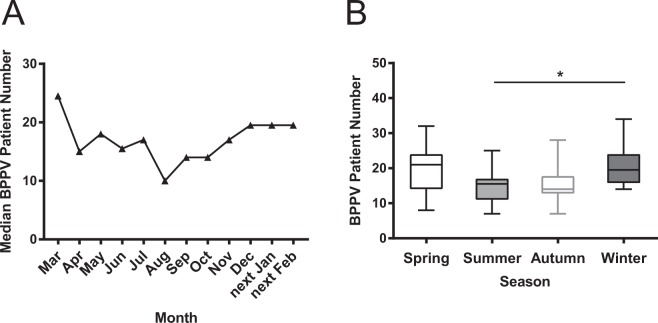
Six-year pooled BPPV patient numbers in each month and four seasons. Notes: (**A**) The graph shows the distribution of six-year pooled BPPV patient numbers per month (in median value). It significantly increased in winter, dramatically decreased in mid spring, reached the lowest numbers in late summer, and increased again in mid-autumn. (**B**) The box plots show that the six-year pooled BPPV patient numbers in winter group (Dec to following Feb) were significantly higher than those in summer group (Jun to Aug). *Indicates *p* < 0.05 and error bars indicate interquartile range. Dec: December; Feb: February; Jun: June; Aug: August.

For the study, 1269 patients were recruited and diagnosed with idiopathic BPPV from March 2013 to February 2019. The demographic, clinical, and biochemical data were collected for final analysis **(**Fig. 1**)**. Among these patients, 942 and 327 patients were identified as having posterior and horizontal canal BPPV, respectively. The median age of all BPPV patients was 60 years (IQR 51–67 years). Nine hundred and Nineteen women were identified; the ratio of men to women was 1:2.6. The median time period between symptom onset to confirmed diagnosis in our center was 7 days (IQR 4–14 days). Our results found that December to next March were the top 4 months with more BPPV patients. The median BPPV patient numbers per month declined dramatically in mid-spring, reached the lowest numbers in late summer, and increased again in mid-autumn **(**Fig. 2A**)**. The six-year pooled BPPV patient numbers of winter group were significantly higher than those of summer group [20 (IQR 16–24) vs. 16 (IQR 11–17) patients, *p* < 0.05, 95% CI (−11.15, −0.63)] **(**Fig. 2B**)**. There seems to be a trend that the BPPV patient numbers in winter group were higher than those in autumn group as well [20 (IQR 16–24) vs. 14 (IQR 13–18) patients, *p* = 0.089, 95% CI (−0.42, 10.09)]. Furthermore, neither age nor gender was significantly different among four groups in the entire cohort (*p* > 0.05) (Supplementary Figs S1, S2 and Table S3**)**.

### 25-hydroxyvitamin D levels

**Figure 3:**
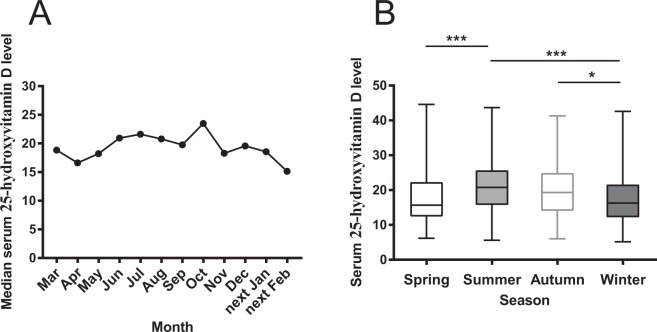
Six-year pooled serum 25-hydroxyvitamin D level of BPPV patient in each month and four seasons. Notes: (**A**) The graph shows the distribution of serum 25-hydroxyvitamin D levels of BPPV patients (in median value) registered during the time period from March 2013 to February 2019. They slowly increased in mid-spring, reached a peak in mid-autumn, and quickly declined in late autumn. (**B**) The box plots show that the serum 25-hydroxyvitamin D values of BPPV patients in winter group (Dec to following Feb) were much lower than those in summer (Jun to Aug) and autumn group (Sep to Nov). In addition, values of BPPV patient in spring group were significantly lower than those in summer group as well. It seems to be a trend that vitamin D values in spring group were also lower than those in autumn group (*p* = 0.06). ***Indicates *p* < 0.001, *indicates *p* < 0.05. Error bars indicate interquartile range. Dec: December; Feb: February; Jun: June; Aug: August; Sep: September; Nov: November.

**Figure 4:**
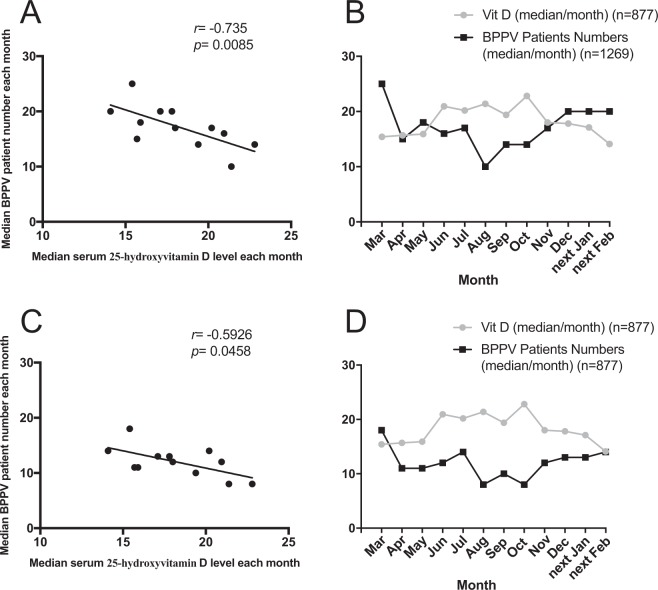
Correlation between BPPV patient numbers and serum 25-hydroxyvitamin D levels. Notes: (**A**) The scatter dot plot illustrates a moderate, negative correlation between median six-year pooled BPPV patient numbers per month (n = 1269) and serum 25-hydroxyvitamin D levels (n = 877). (**B**) The curve shows the merged variation of median six-year pooled BPPV patient numbers per month and serum 25-hydroxyvitamin D levels. (**C**) The scatter dot plot shows a moderate, negative correlation between median BPPV patient numbers (n = 877) for whom vitamin D values were available and serum 25-hydroxyvitamin D levels (n = 877) in six years. (**D**) The curve shows the merged variation of median BPPV patient numbers with vitamin D values and serum 25-hydroxyvitamin D levels in six years.

Among the entire cohort, serum 25-hydroxyvitamin D values were available for 877 patients. The data showed that these levels slowly increased per month in mid-spring, reached a peak in mid-autumn, and quickly declined in late autumn **(**Fig. 3A**)**. The serum 25-hydroxyvitamin D levels in patients of winter group were much lower than those of summer and autumn group [16.3 (IQR 12.2–21.6) vs. 20.8 (IQR 15.7–25.7) ng/ml, *p* < 0.001; 16.3 (IQR 12.2–21.6) vs. 19.3 (IQR 14–25) ng/ml, *p* < 0.05, respectively] **(**Fig. 3B**)**. In addition, levels in BPPV patients of spring group were also significantly lower than those of summer group [15.7 (IQR 12.4–22.3) vs. 20.8 (IQR 15.7–25.7) ng/ml, *p* < 0.001]. It seems to be a trend that vitamin D values in spring group were also lower than those in autumn group *(p* = 0.06). A moderate negative correlation was found between six-year pooled BPPV patient numbers per month and serum 25-hydroxyvitamin D levels (*r* = −0.735, *p* = 0.0085 (Fig. 4A,B). A moderate negative correlation was also found between BPPV patient numbers whose vitamin D values were available and median serum 25-hydroxyvitamin D levels for six years (*r* = −0.5926, *p* = 0.0458) (Fig. 4C,D).

## Discussion

To our knowledge, this is the first study in China to investigate whether seasonal variation possibly represents a relevant factor for the occurrence of idiopathic BPPV in 1269 patients. Based on a six-year retrospective registry, the main important results are twofold. (1) As reported in other countries, the onset of BPPV in Shanghai China may also have a seasonality. More people present with BPPV in winter than those in summer. (2) The seasonal variation of BPPV may correlate with serum vitamin D level fluctuation.

With respect to seasonality of BPPV onset, the present study is the fourth report worldwide. Methodologically, the first report of this phenomenon comes from Whitman and coworkers in the USA^10^, BPPV diagnosis is based on medical record, which is still different from face to face interview with positive positional evoked maneuvers. Patients from all the other three studies (USA, UK and Iraq) were from ENT clinic or Eye and Ear Infirmary, only patients from the present study comes from vertigo clinic of Neurology department with available vitamin D level (Table 1). Regarding our main results, March is a consistent month with high BPPV patient numbers, indicating that there might be a seasonality of BPPV. However, none of previous studies undertook further seasonal subgroup comparison in their patients. Our results showed that December to next March (winter and early spring) are the main months with higher diagnosed BPPV numbers. Further sub-grouping comparison demonstrated that winter had more BPPV incidence than summer (Fig. 2).

**Table 1:** Serial epidemiological studies regarding seasonality of BPPV.

Author, Year	City, Country	Sample size	Source	BPPV diagnosis	Month with high BPPV numbers	Vitamin D level
Whitman GT, *et al*. 2015	Boston, USA	956	Eye and Ear Infirmary	medical record	Mar to May	not available
Saeed BMN, *et al*. 2016	Duhok, Iraq	207	ENT clinic	positive positional evoked maneuver	Mar	not available
Meghji S, *et al*. 2017	Norwich, UK	339	ENT clinic	positive positional evoked maneuver	Mar to May	not available
The present study	Shanghai, China	1269	Neurology vertigo clinic	positive positional evoked maneuver	Dec to Next Mar	available

Note: Mar: March; Dec: December.

We also found that there was a seasonal variation in vitamin D levels in 877 BPPV patients and a moderate, negative correlation between patient numbers and serum vitamin D level, indicating that low vitamin D levels in winter may constitute a risk factor for the increasing incidence of BPPV in this period. In addition to dietary means, vitamin D is mainly synthesized in the skin with sufficient levels of sunlight containing ultraviolet radiation. Its biological form is 1,25 dihydroxy vitamin D after being hydroxylated in liver and kidney. Serum 25-hydroxyvitamin D is a well-known marker to determine a subject’s vitamin D status^16,17^. Compared with summer, there was less sunlight time and a lower ultraviolet index, leading to decreased vitamin D levels in winter and spring, as shown in the national nutrition and health survey in China^18^. Physiologically, vitamin D is beneficial to the upregulation of Ca^2+^ channel transporters in the epithelia of the semicircular canal and utricular otolith, which could maintain the low Ca^2+^ environment in endolymph of the inner ear^19^. Therefore, low vitamin D levels could prompt the formation of calcium carbonate in endolymph. This may explain the relatively high incidence of BPPV in winter. Although a series of clinical observational studies reported that vitamin D levels were decreased in idiopathic BPPV^20^, seasonal factors should also be considered in future related studies.

Our results have important clinical implications. As the incidence of BPPV in our center seems to increase in winter, more medical staffs and resources should be devoted to the vertigo outpatient clinic to deal with the mounting numbers of patients during this period. This study also has a few limitations: selected bias may exist since this is a retrospective study based on one single outpatient vertigo clinic in the neurology department, which will miss some BPPV patients who visited the emergency department or ENT outpatient clinic. In addition, the vitamin D levels are only reported from 69% of BPPV patients and not from healthy controls or disease controls. Therefore, further validated studies from multi-center, cross-sectional registries are needed to verify our pilot results. Whether supplementation of vitamin D will decrease the onset of BPPV in winter merits further investigation.

In summary, the present study demonstrated that there are seasonal variations in BPPV patient numbers and serum vitamin D levels and that a moderate, negative correlation existed between the two variables, based on our six-year registry cohort. Decreased vitamin D levels may represent a related factor for the increased incidence of BPPV in winter and early spring.

## Supplementary information

Seasonal variation of idiopathic benign paroxysmal positional vertigo correlates with serum 25-hydroxyvitamin D levels: a six-year registry study in Shanghai, China

## Data Availability

The datasets generated during and analysed during the current study are available from the corresponding author on reasonable request.
