# Supplementary material for: Seasonal variation of idiopathic benign paroxysmal positional vertigo correlates with serum 25-hydroxyvitamin D levels: a six-year registry study in Shanghai, China
Source: Sci Rep. 2019 Nov 7;9:16230. doi: 10.1038/s41598-019-52803-4 (PMC6838600; doi:10.1038/s41598-019-52803-4)
Supplement: Supplementary file 1 — Seasonal variation of idiopathic benign paroxysmal positional vertigo correlates with serum 25-hydroxyvitamin D levels: a six-year registry study in Shanghai, China [file 41598_2019_52803_MOESM1_ESM.docx]

**Supplementary Information**

**Seasonal variation of idiopathic benign paroxysmal positional vertigo correlates with serum 25-hydroxyvitamin D levels: a six-year registry study in Shanghai, China**

Liang Shu^†1^, Jing Wu^†1^, Chun-Yan Jiang^†1^, Xu-Hong Sun^1^, Hui Pan^1^, Jie Fang^1^, Yi Tang^2^, Si-Cheng Wu^3^, Jian-Ren Liu^*1^, Wei Chen^*1^

^1^ *Department of Neurology, Shanghai Ninth People’s Hospital, Shanghai Jiao Tong University School of Medicine, Shanghai, China*

^2^ *Department of Clinical Laboratory, Shanghai Ninth People’s Hospital, Shanghai Jiao Tong University School of Medicine, Shanghai, China*

^3^ *Biostatistics Office of Clinical Research Center, Shanghai Ninth People’s Hospital, Shanghai Jiao Tong University School of Medicine, Shanghai, China*

**Supplementary Fig. S1. Age of six-year pooled BPPV patients among four groups.**

**Notes: The histograms show there are no significant differences among four groups in six-year pooled BPPV patients regarding age (in median value) (*p*>0.05).**

**Supplementary Fig S2. Gender of six-year pooled BPPV patients among four groups.**

**Notes: The graph shows female BPPV patients in our cohort are much more than male patients.**

**Supplementary Table S3. Differences among four groups in six-year pooled BPPV patients regarding gender.**

|  | Spring | Summer | Autumn | Winter | *p* value ^$^ |
| --- | --- | --- | --- | --- | --- |
| Male | 98 | 65 | 92 | 95 | 0.141 |
| Female | 248 | 201 | 192 | 278 |  |

**Notes: There is no significant difference in six-year pooled BPPV patients between each two groups with regard to gender.**

**^$^ *p* values was calculated by Fisher’s exact test (two-tailed).**
